# Evidence of a liquid–liquid phase transition in H$_2$O and D$_2$O from path-integral molecular dynamics simulations

**DOI:** 10.1038/s41598-022-09525-x

**Published:** 2022-04-09

**Authors:** Ali Eltareb, Gustavo E. Lopez, Nicolas Giovambattista

**Affiliations:** 1 Department of Physics, Brooklyn College of the City University of New York, Brooklyn, New York 11210 USA; 2 Department of Chemistry, Lehman College of the City University of New York, Bronx, NY 10468 USA; 3 Ph.D. Program in Physics, The Graduate Center of the City University of New York, New York, NY 10016 USA; 4 Ph.D. Program in Chemistry, The Graduate Center of the City University of New York, New York, NY 10016 USA

**Keywords:** Chemistry, Physics

## Abstract

We perform path-integral molecular dynamics (PIMD), ring-polymer MD (RPMD), and classical MD simulations of H$_2$O and D$_2$O using the q-TIP4P/F water model over a wide range of temperatures and pressures. The density $\rho (T)$, isothermal compressibility $\kappa _T(T)$, and self-diffusion coefficients *D*(*T*) of H$_2$O and D$_2$O are in excellent agreement with available experimental data; the isobaric heat capacity $C_P(T)$ obtained from PIMD and MD simulations agree qualitatively well with the experiments. Some of these thermodynamic properties exhibit anomalous maxima upon isobaric cooling, consistent with recent experiments and with the possibility that H$_2$O and D$_2$O exhibit a liquid-liquid critical point (LLCP) at low temperatures and positive pressures. The data from PIMD/MD for H$_2$O and D$_2$O can be fitted remarkably well using the Two-State-Equation-of-State (TSEOS). Using the TSEOS, we estimate that the LLCP for q-TIP4P/F H$_2$O, from PIMD simulations, is located at $P_c = 167 \pm 9$ MPa, $T_c = 159 \pm 6$ K, and $\rho _c = 1.02 \pm 0.01$ g/cm$^3$. Isotope substitution effects are important; the LLCP location in q-TIP4P/F D$_2$O is estimated to be $P_c = 176 \pm 4$ MPa, $T_c = 177 \pm 2$ K, and $\rho _c = 1.13 \pm 0.01$ g/cm$^3$. Interestingly, for the water model studied, differences in the LLCP location from PIMD and MD simulations suggest that nuclear quantum effects (i.e., atoms delocalization) play an important role in the thermodynamics of water around the LLCP (from the MD simulations of q-TIP4P/F water, $P_c = 203 \pm 4$ MPa, $T_c = 175 \pm 2$ K, and $\rho _c = 1.03 \pm 0.01$ g/cm$^3$). Overall, our results strongly support the LLPT scenario to explain water anomalous behavior, independently of the fundamental differences between classical MD and PIMD techniques. The reported values of $T_c$ for D$_2$O and, particularly, H$_2$O suggest that improved water models are needed for the study of supercooled water.

## Introduction

Water is an anomalous liquid with thermodynamic and dynamical properties that behave unexpectedly upon cooling and/or pressurization; see, e.g., Ref.^1^. For example, experiments performed in the 1970’s by Angell et al.^2–4^ show that water isobaric heat capacity $C_P(T)$ and isothermal compressiblity $\kappa _T(T)$ exhibit an apparent divergency at $T\approx 228$ K and $P = 0.1$ MPa. More recent experiments that extend Angell’s studies to lower temperatures identify a maxima in $C_P(T)$ and $\kappa _T(T)$ at $T \approx 228$ K ($P = 0.1$ MPa)^5,6^. Although many theoretical approaches have been proposed to explain water anomalous behavior, the so-called liquid–liquid phase transition (LLPT) scenario^7,8^ is currently the explanation best-supported by experiments^5,9–13^, computer simulations^7,14–18^, and theory^14,19–23^. In the LLPT scenario, water at low temperatures exists in two distinct liquid states, low-density and high-density liquid (LDL and HDL). In the P–T plane, LDL and HDL are separated by a first-order LLPT line that ends at a liquid–liquid critical point (LLCP) at higher temperatures. Importantly, the LLPT hypothesis explains naturally the maxima in $\kappa _T(T)$ and $C_P(T)$ observed recently upon cooling water at $P=0.1$ MPa^5,10^. It also explains, naturally, the complex behavior of water in the glass state^8,9,24–34^ which, arguably, is not clearly explained by other approaches, such as the singularity free scenario^35^.

Available experimental data suggest that the LLCP in water is located at about $P_c$ = 50–100 MPa and $T\approx 220$ K^1,12^. Unfortunately, due to water rapid crystallization at these conditions, the existence of the LLCP in water has not been confirmed in experiments. Strong evidence for the existence of a LLPT in water is available from recent sub-microsecond experiments at $T\approx 205$ K^9^; additional evidence of the LLPT in water is available from experiments performed at $T\approx$ 130–140 K, in the the so-called ultraviscous liquid state of water^36,37^. Many computer simulations validate the LLPT hypothesis. Specifically, a LLCP has been identified in classical computer simulations using popular models, such as ST2, TIP4P/2005, and TIP4P/ice^7,14,18,38–44^. A recent classical MD simulation using a water model developed from density functional theory combined with machine learning techniques also suggests that water exhibits a LLCP in the supercooled regime^21^. Not surprisingly, the location of the LLCP in computer simulations vary with the water model considered. For example, in the case of the ST2 water model, the LLCP temperature is overestimated ($T_c=237$ K, $P_c=167$ MPa, $\rho _c =0.99$ g/cm$^3$)^41^; while in the TIP4P/2005 and TIP4P/Ice water models it is underestimated ($T_c=172$ K, $P_c=186$ MPa, and $\rho _c=1.03$ g/cm$^3$ for TIP4P/2005; $T_c=188$ K, $P_c=175$ MPa, $\rho _c=1.01$ g/cm$^3$ for TIP4P/Ice)^18^. In all these cases, the LLCP pressure is overestimated by approximately 100 MPa^6^. The computer simulation studies that find a LLCP in water are based on classical models where the atoms delocalization due to nuclear quantum effects (NQE) are neglected. This can be troublesome because water is a light molecule and delocalization effects of its H atoms occur even at standard temperatures and pressures^45,46^. For example, the temperature of maximum density and the glass transition temperature (T$_g$) of H$_2$O and D$_2$O differ by 7–10 K, a clear sign of non-negligible NQE. Path-integral computer simulations of water-like models that have a LLCP clearly show that NQE can indeed shift the location of the LLCP as well as alter the shape and slope of the $C_P(T)$ and $\kappa _T(T)$ maxima lines^47,48^. Interestingly, while experiments in glassy water have estimated differences in the location of the LLCP in H$_2$O and D$_2$O ($\Delta T_c \approx 10$ K, $\Delta P_c \approx 50$ MPa)^49,50^, the issue of isotope effects on the location of the LLCP has not been explored in computational and theoretical studies.

In this work we perform extensive path-integral, ring-polymer, and classical molecular dynamics (PIMD, RPMD, MD) simulations of light and heavy water using the q-TIP4P/F model and explore the corresponding phase diagram and thermodynamic/dynamical anomalous properties. One goal of this work is to determine the NQE (due to atoms delocalization) on the location of the LLCP, LLPT, and supercritical anomalous lines (such as maxima lines in $\rho$, $C_P$, and $\kappa _T$) in q-TIP4P/F water (H$_2$O). The second goal of this work is to study isotope substitution effects in water, i.e., whether PIMD simulations of H$_2$O and D$_2$O can reproduce the subtle differences in the phase diagram and anomalous properties of H$_2$O and D$_2$O observed in experiments. In a previous study, we performed PIMD simulations of q-TIP4P/F water at $P=0.1$ MPa and showed that this model reproduces some signatures of the LLPT scenario, specifically, a maximum in $\kappa _T(T)$ was found in H$_2$O and D$_2$O at $T \approx$ 230–235 K ($P = 0.1$) MPa^46^. Here, we extend our previous study to a wide range of temperatures and pressures in order to explore the possible existence of a LLCP in H$_2$O and D$_2$O. By combining the PIMD/MD results and the two-state equation of state (TSEOS)^14,22,23^, we are able to identify a LLCP in both H$_2$O and D$_2$O. The TSEOS is based on the assumption that liquid water is a mixture of two interconvertible (liquid) states. The TSEOS has been shown to fit remarkably well the computer simulations results obtained from classical MD simulations of ST2 and TIP4P/2005 water as well as a water model based on DFT and machine learning techniques^14,19–21,23^; it has also been applied to the case of real water^20,51^. While at low temperatures the TSEOS predicts that water separates into LDL and HDL, at high temperatures ($T > 270$ K), it predicts a rather homogeneous liquid (HDL) which is consistent with recent computer simulations^52,53^.

Our paper is organized as follows. In “Simulation method”, we present the computer simulation details. In “Results”, we discuss the results from our PIMD/RPMD and classical MD simulations of H$_2$O using the q-TIP4P/F water model. The phase diagram of D$_2$O is briefly discussed and compared with the phase diagram of H$_2$O. A summary and discussions are included in “Summary and discussion”.

## Simulation method

Our results are based on PIMD/RPMD and classical MD simulations of a system composed of $N = 512$ water molecules in a cubic box with periodic boundary conditions. H$_2$O and D$_2$O molecules are represented using the non-rigid q-TIP4P/F model^54^. This model is based on the TIP4P/2005 model for water^55^, commonly used in classical MD simulations. The q-TIP4P/F water model was optimized for path integral computer simulations and has been shown to be able to reproduce remarkably well the properties of liquid water at $P=~0.1$ MPa^46,54^. Here, we perform PIMD and MD simulations at constant *N*, *P*, and *T* over a wide range of temperatures and pressures, $180 \le T \le 375$ K and $-250 \le P \le$ 500 MPa; see Supplementary Fig. S1 of the Supplementary Information (SI). The temperature of the system is maintained constant using a stochastic (local) path integral Langevin equation (PILE) thermostat^56^ while the pressure of the system is controlled by using a Monte Carlo Barostat (additional computational details can be found in Ref.^46^). In the PIMD simulations, the time step *dt* is set to 0.25 fs and the number of beads per ring-polymer/atom was set to $n_b=32$; in Ref.^46^, it is shown that this value of $n_b$ is large enough to obtain well-converged dynamical, thermodynamic, and structural properties of q-TIP4P/F water at $P=0.1$ MPa and $T \ge 210$ K. In order to ensure that the conclusions in Ref.^46^ applied to the pressures we considered in this work, we have also performed additional PIMD simulations using $n_b = 72$ beads per ring-polymer (see SI). Consistent with Ref.^46^, we found that most of the thermodynamic and dynamical properties converged with $n_b = 32$, with the enthalpy being the only expected exception. Short-range (Lennard–Jones pair potential) interactions are calculated using a cutoff $r_c$ = 1.0 nm and long range electrostatic interactions are computed using the Particle Mesh Ewald (PME) method with the same cutoff $r_c$. In the classical MD simulations, we employ a time step $dt=0.50$ fs and set $n_b=1$. All PIMD and classical MD simulations are performed using the OpenMM software package (version 7.4.0)^57^. The OpenMM software package is also used to perform the RPMD simulations which are used for the calculation of the diffusion coefficients of H_2_O and D_2_O. Details on the calculation of the diffusion coefficients can be found in Ref.^46^. We note that in the OpenMM software package, the RPMD application sets the mass of the ring-polymer beads to the physical mass of the corresponding atom. When used to calculate static equilibrium properties (energies, density, and RDF), the RPMD simulations reduce to PIMD simulations.

In all PIMD/RPMD and classical MD simulations, the system is equilibrated for a time interval $t_{eq}$ followed by a production run of time length $t_{prod}$. The values of $t_{eq}$ and $t_{prod}$ depend on the state point simulated. Typical simulation times for PIMD/RPMD range from 2.5 ns to 100 ns. Simulation times for classical MD simulations range from 2.5 ns to 2–3 $\upmu s$. To confirm that the system reaches equilibrium, we monitor the mean-square displacement (MSD) of the water molecules in the system as a function of time and confirm that the PIMD/RPMD and classical MD simulations satisfy the requirement that $t_{eq}$, $t_{prod} > \tau$, where $\tau$ is the time it takes for the MSD of water molecules to reach 1 nm$^2$.

## Results

The results are presented as follows. In “Liquid–liquid phase transition”, we show that the phase diagrams of H$_2$O from MD and PIMD simulations are consistent with the existence of a LLPT and LLCP at low temperatures. Since a LLCP generates anomalous loci of maxima in $C_P$ and $\kappa _T$, the behavior of $C_P(T)$ and $\kappa _T(T)$ are discussed in “[Sec Sec5]”. The self-diffusion coefficient of H$_2$O and D$_2$O are the topic of “Diffusion coefficient” where we identify the anomalous locus of diffusivity maxima. A complete phase diagram for H$_2$O is presented in “Phase diagram” where we also discuss similar results obtained for D$_2$O.

### Liquid–liquid phase transition

**Figure 1:**
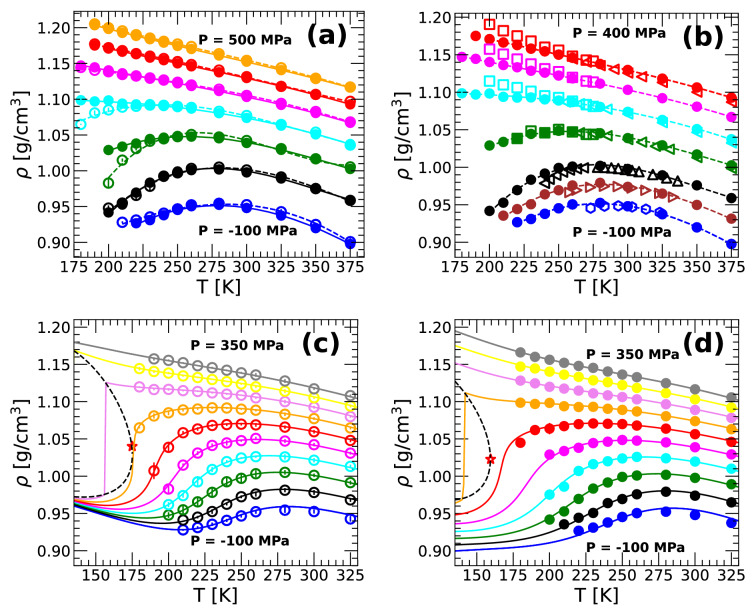
Density of q-TIP4P/F water as function of temperature along selected isobars. (**a**) Comparison of $\rho (T)$ from classical MD (open circles) and PIMD simulations (solid circles). Pressures are (bottom to top) $P={-100},~0.1,~100,~200,~300,~400,~500$ MPa. (**b**) Density of q-TIP4P/F water from PIMD simulations (solid circles) and experiments (open symbols; squares, left-triangles, up-triangles, right-triangles, and diamonds are, respectively, from Refs.^12,16,59–61^). Pressures are (bottom to top) $P={-100},~{-50},~0.1,~100,~200,~300,~400$ MPa. Deviations between experiments and simulations are noticeable only at high pressures, $P>200$ MPa, and low temperatures, $T<240$ K. (**c**) Fit of the q-TIP4P/F water densities shown in (**a**) using the TSEOS (solid lines). (**d**) Fit of the q-TIP4P/F water densities shown in (**b**) using the TSEOS (solid lines). Pressures in (**c**) and (**d**) are (bottom to top) $P = ~{-100},~{-50},~0.1,~50,~100,~150,~200,~250,~300,~350$ MPa. The liquid–liquid binodal line and LLCP predicted from the TSEOS are denoted by the black dashed line and red star.

Figure 1a shows the density of H$_2$O from both classical MD (open circles) and PIMD simulations (solid circles) along the isobars $P = -100,~0.1,~100, \ldots ,~500$ MPa and at temperatures in the range $T =$ 180–375 K. The densities from MD and PIMD simulations overlap practically throughout the entire temperature and pressure range considered with some deviations being noticeable only at $P=$ 100–200 MPa and $T<240$ K. As we will show below, these T–P conditions are in the proximity of the LLCP. We note that the densities of q-TIP4P/F H$_2$O are in remarkable good agreement with the corresponding experimental values. To show this, we include in Fig. 1b the densities from experiments and PIMD simulations of q-TIP4P/F H$_2$O. Deviations between experiments and PIMD simulations are small, $\Delta \rho<$ 0.02–0.03 g/cm$^3$, and are present only at $P > 200$ MPa and $T <250$ K (similar values of $\Delta \rho$ hold for the case of MD simulations). It follows that both MD and PIMD simulations of q-TIP4P/F water predict the correct location (T and P) for the density maximum of water. In these and other cases, the computer simulation results can be fitted remarkably well using the TSEOS^14,20–23,58^. In Fig. 1c,d, we compare the $\rho (T)$ isobars obtained from the MD and PIMD simulations of q-TIP4P/F water with the corresponding fit using the TSEOS (a brief explanation of the methodology used to obtain the TSEOS can be found in Refs.^14,21^). The TSEOS is fitted using only the PIMD and MD data for $180 \le T \le 325$ K and $-50 \le P \le 350$ MPa. As shown in Fig. 1c,d, the TSEOS isobars are in excellent agreement with the simulation results over a majority of the state points simulated. Interestingly, the TSEOS also predicts a minimum in the $\rho (T)$ isobars of q-TIP4P/F water. While at the studied temperatures we do not observe density minima in the classical MD and PIMD simulations, density minima were reported at different pressures in TIP4P/2005 and ST2 water^15,42^. The optimized parameters for the TSEOS are given in Table S1 of the SI.

The TSEOS also provides a good estimation of the LLCP location. For example, in the case of TIP4P/2005 water, the TSEOS predicts that $T_c = 182$ K, $P_c = 170$ MPa, and $\rho _c = 1.02$ g/cm$^3$^14^, which is in good agreement with recent MD simulations that were able to access the LLCP, $T_c = 172$ K, $P_c = 186$ MPa, and $\rho _c=1.03$ g/cm$^3$^18^; similar results were found in an MD simulation study combined with the potential energy landscape theoretical approach^43^. Using the TSEOS, one can estimate the LLCP location ($\rho _c,~T_c,~P_c$). The values of ($\rho _c,~T_c,~P_c$) for the case of q-TIP4P/F H$_2$O, based on classical MD and PIMD simulations, are given in Table 1 and are indicated in Fig. 1c,d by a red star. It follows that including NQE can shift the location of the LLCP. Specifically, relative to the classical case (MD simulations), adding NQE (PIMD simulations) lowers $T_c$ and $P_c$ by $16 \pm 6$ K, $36 \pm 10$ MPa, respectively; $\rho _c$ is not affected by the inclusion of NQE. Interestingly, recent studies based on water-like monoatomic model liquids that exhibit a LLCP, show that including NQE has the effects of lowering $T_c$ and increasing $P_c$, while leaving $\rho _c$ unaffected^47,48^.

**Table 1:** Estimated pressure $P_c$, temperature $T_c$, and density $\rho _c$ of the LLCP of q-TIP4P/F H$_2$O. Values of $P_c$, $T_c$, and $\rho _c$ are obtained by using the TSEOS in combination with data from classical MD and PIMD simulations at $180\le T \le 325$ K. Numbers in parenthesis are standard deviations.

	PIMD	Classical MD
$P_c$	167 (9)	203 (4)
$T_c$	159 (6)	175 (1)
$\rho _c$	1.02 (0.01)	1.03 (0.01)

**Figure 2:**
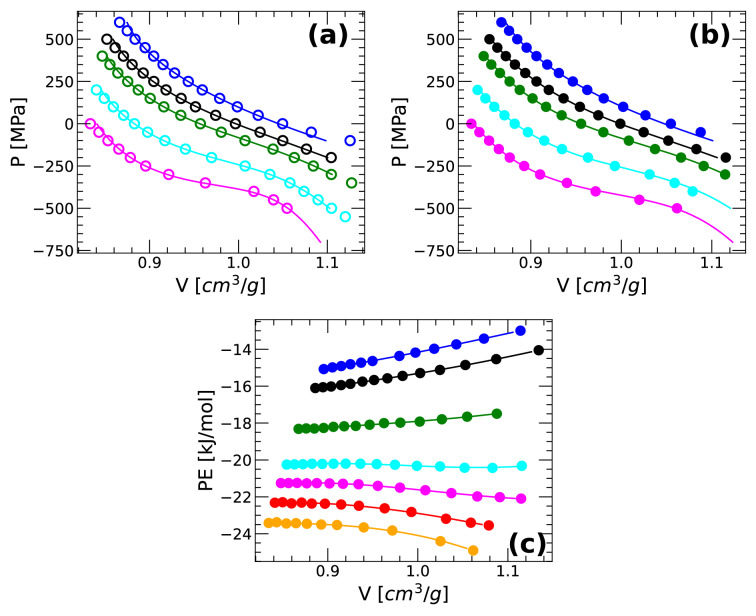
Pressure of q-TIP4P/F water as a function of volume along selected isotherms. Circles are results from (**a**) classical MD and (**b**) PIMD simulation. The solid lines are the results from the TSEOS. Isotherms correspond to (top to bottom) $T=~300,~260,~240, 220, 200$ K and are shifted by $\delta P=~100,~0,~-100,~-300,~-500$ MPa, respectively. An inflection point in *P*(*V*) seems to develop at $T < 200$ K consistent with the existence of a LLPT in q-TIP4P/F water at lower temperatures. (**c**) Potential energy for selected isotherms at (bottom to top) $T=~200,~220,~240,~260,~300,~350,~375$ K. Solid symbols are from PIMD simulations; lines are guides to the eye.

We also compare the volumes predicted by the TSEOS with the corresponding values obtained from our MD and PIMD simulations. Fig. 2a,b show *P*(*V*) along isotherms based on the classical MD and PIMD simulations, respectively. In both cases, MD and PIMD simulations, the values of *P*(*V*) obtained from the TSEOS are in excellent agreement with our simulations. This strongly indicates that the TSEOS is reliable in predicting the properties of q-TIP4P/F water from both MD and PIMD simulations. We note that the *P*(*V*) isotherms shown in Fig. 2a,b seem to develop an inflection point as the temperature decreases, consistent with the existence of a LLCP at $T<200$ K. Similarly, as shown in Fig. 2c, the potential energy *PE*(*V*) along isotherms is an increasing function of *V* at high temperatures but it develops a concave region (i.e., $\left( \partial ^2 PE/\partial V^2 \right) _{N,T}<0$) at low temperatures. The Helmholz free energy of the system is $F(N,V,T)=E-TS$ and hence, a concavity in *PE* can lead to a concavity in *F*(*V*) (at constant N and T) at very low temperatures. A concavity in *F*(*V*) implies that the system exhibits a first-order (liquid–liquid) phase transitions^62^, again, consistent with the presence of LLCP/LLPT at low temperatures.

### Thermodynamic response functions: $\kappa _T$ and $C_p$

**Figure 3:**
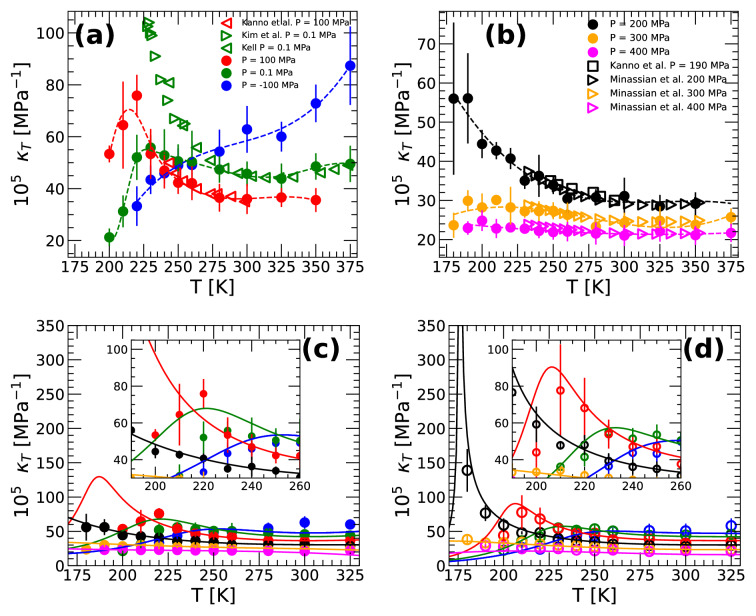
(**a**) Isothermal compressibility of q-TIP4P/F water from PIMD simulations at pressures $P=-100,~0.1,100$ MPa (solid circles; dashed lines are guides to the eye). Experimental data for $\kappa _T(T)$ are indicated by open symbols (red left-triangles, green left- and right-triangles are from Refs.^5,65,66^, respectively). (**b**) Same as (**a**) for pressures $P=~200,~300,~400$ MPa; experimental values of $\kappa _T(T)$ are from Refs.^65,67^. (**c**) Comparison between the values of $\kappa _T(T)$ obtained from the PIMD simulations [solid circles; from (**a**) and (**b**)] and the TSEOS (solid lines). (**d**) Comparison between the values of $\kappa _T(T)$ obtained from the MD simulations (empty circles) and the TSEOS (solid lines). Insets are magnifications of the main panels. The predictions of the TSEOS are in very good agreement with the MD simulation results and semiquantitative agreement in the case of PIMD simulations.

We obtain the isothermal compressibility of q-TIP4P/F water by calculating the density fluctuations of the system^63^,

1 $$\begin{aligned} \kappa _T(T) = \frac{\langle V^2 \rangle - \langle V \rangle ^2}{k_B T \langle V \rangle }, \end{aligned}$$

where $\langle \ldots \rangle$ indicates average over time and $k_B$ is the Boltzmann’s constant. Fig. 3a,b show the $\kappa _T(T)$ for H$_2$O obtained from PIMD simulations (solid circles) together with available experimental data (open symbols) at low and high pressures, respectively. At $P\ge 200$ MPa (Fig. 3b), the experimental and PIMD simulation values of $\kappa _T(T)$ practically overlap; a similar agreement is found at $P=100$ MPa (Fig. 3a). However, at $P=0.1$ MPa, where more experimental data is available, the experimental $\kappa _T(T)$ increases more rapidly upon cooling than found in PIMD simulations. Hence, relative to real water, the density fluctuation in q-TIP4P/F water are underestimated at $P=0.1$ MPa and in the supercooled regime. We note that the values of $\kappa _T(T)$ obtained from classical MD and PIMD simulations overlap (within error bars) at $T \ge 190$ K and hence, they were omitted in Fig. 3a,b; the $\kappa _T(T)$ obtained from MD simulations is shown in Fig. S2 of the SI.

An important result from Fig. 3a is the presence of maxima in $\kappa _T(T)$ at $P=0.1$ and 100 MPa. This is an anomalous property that was originally predicted by the LLPT hypothesis scenario and later confirmed by experiments^5,17^. The experimental data from Ref.^5^ is included in Fig. 3a; the experimental $\kappa _T$-maximum occurs at $T=228$ K ($P=0.1$ MPa; open right triangles) and it is very sharp. While the $\kappa _T$-maximum in PIMD simulations occurs at a similar temperature ($T=230$ K), this maximum is much smaller and wider relative to the experiments. Within the LLCP hypothesis scenario, the $\kappa _T$-maximum is expected to increase as one approaches the LLCP and it should diverge at the LLCP. This is fully consistent with the PIMD simulations results shown in Fig. 3a. Specifically, as the pressure increases from $P=0.1$ MPa to $P=100$ MPa, the $\kappa _T$-maximum shifts to lower temperatures and increases in height. The same behavior of $\kappa _T$ is found in classical MD simulations of water models that exhibit a LLCP^14,15,21,64^.

We also calculate $\kappa _T(T)$ using the TSEOS. The TSEOS provides an expression for the Gibbs free energy of the system from which the isothermal compressibility can easily be obtained,

2 $$\begin{aligned} \kappa _T(T) = -\frac{\left( \partial ^2 G/ \partial P^2\right) _T}{\left( \partial G / \partial P\right) _T}. \end{aligned}$$

A comparison of the values of $\kappa _T(T)$ obtained from the TSEOS and our MD/PIMD simulations are presented in Fig. 3c,d. The predictions from the TSEOS agree rather well with the MD simulation results [inset of Fig. 3d]. In the case of PIMD simulations [inset of Fig. 3c], the TSEOS provides compressibility values that are in good agreement with the simulation results at high temperatures. However, at lower temperatures, the TSEOS predicts slightly larger maxima in $\kappa _T$ that are shifted to lower temperatures relative to the simulations. This suggests that, the location of the LLCP in q-TIP4P/F water from PIMD may be located at slightly lower $T_c$ and/or higher $P_c$ relative to the corresponding *estimated* values resulting from the TSEOS.

Next, we discuss the isobaric heat capacity,

3 $$\begin{aligned} C_P(T) = \left( \frac{\partial H(T)}{\partial T}\right) _{N,P}. \end{aligned}$$

In our previous work (at $P=0.1$ MPa)^46^, the enthalpy was calculated directly from MD and PIMD simulations at selected temperatures and then, the values of *H*(*T*) were fitted using a fourth-order polynomial. The resulting analytic expression for *H*(*T*) was then used in Eq. (3) to calculate $C_P(T)$. The use of a fourth-order polynomial in the fitting procedure is rather arbitrary. It captures the qualitative increase of $C_P(T)$ upon cooling at low pressures but it may play a relevant role in identifying a $C_P$-maximum, which is known to occur in experiments^10^. Accordingly, in this work, we take advantage of the TSEOS and use it to calculate *H*(*T*) at selected pressures; after all, the TSEOS reproduces very well the behavior (and maxima) of $\rho (T)$ (see Fig. 1) and $\kappa _T(T)$ (see Fig. 3). Specifically, for a given pressure, we use the polynomial expression of *G*(*T*) given by the TSEOS and obtain an analytical expression for *H*(*T*) using the Gibbs–Helmholtz equation,

4 $$\begin{aligned} H(T) = - T^2 \left( \frac{\partial (G/T)}{\partial T}\right) _P. \end{aligned}$$

**Figure 4:**
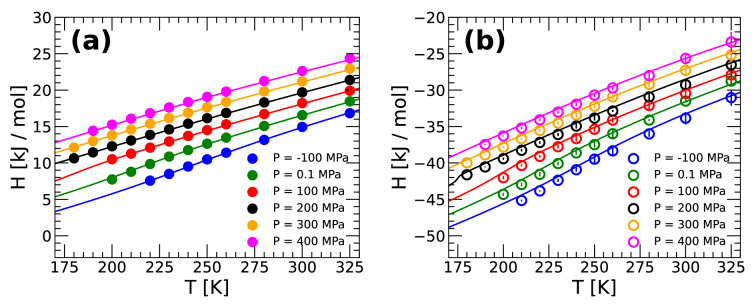
Enthalpy *H*(*T*) of q-TIP4P/F water as a function of temperature for selected pressures. Results are from (**a**) PIMD simulations (solid circles) and (**b**) classical MD simulations (empty circles). Lines are the corresponding *H*(*T*) obtained from the TSEOS. In both cases, the TSEOS predictions are in excellent agreement with the MD/PIMD simulation results.

The obtained *H*(*T*) is then used in Eq. (3) to calculate $C_P(T)$. Figure 4a,b show the *H*(*T*) of q-TIP4P/F water obtained from (i) the TSEOS (solid lines) and (ii) classical MD and PIMD simulations (empty/solid circles). The TSEOS predictions are in excellent agreement with our simulations throughout the entire temperature and pressure range considered in this work.

**Figure 5:**
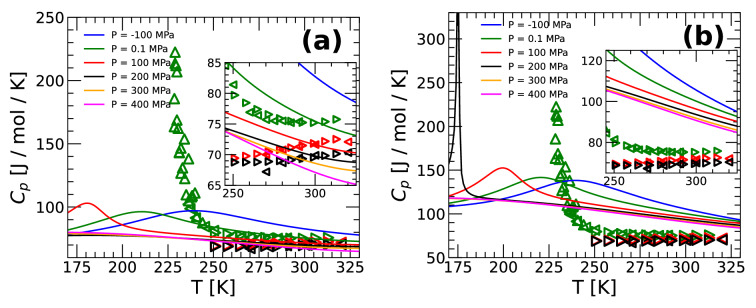
(**a**) Heat capacity $C_P(T)$ of q-TIP4P/F water for P = -100, 0.1, 100, 200, 300, and 400 MPa. $C_P(T)$ was calculated by using Eq.  (3) and the *H*(*T*) expression obtained from the TSEOS and PIMD simulations (solid lines). Experimental data are indicated by empty triangles (left-triangles from Refs.^4,69^; right-triangles from Refs.^59^). (**b**) Same as (**a**) for the case of classical MD simulations. The experimental data from Pathak et al.^10^ (green up-triangles) show a maximum at $T \approx 228$ K.

Figure 5a,b show, respectively, the $C_P(T)$ of q-TIP4P/F water from PIMD and classical MD simulations at selected pressures, above and below the estimated LLCP pressure; open symbols are experimental values. The $C_P(T)$ from classical MD and PIMD simulations are qualitatively similar. Specifically, at the temperature studied, $C_P(T)$ exhibits a maximum at approximately $P\le 200$ MPa. This $C_P$-maximum increases and shifts to lower temperatures as the pressure increases towards the LLCP pressure. At $P > 200$ MPa $> P_c$, $C_P(T)$ is a monotonic decreasing function of *T*. Note that classical MD simulations predict much larger values of $C_P(T)$ than found in PIMD simulations (which is known to occur when NQE are omitted^68^).

Differences between the experimental data and MD/PIMD simulations are noticeable. For example, as shown in Fig. 5a, at $P \ge 100$ MPa, PIMD simulations predict that $C_P(T)$ decreases upon heating while experiments show the opposite behavior. In particular, at $P=0.1$ MPa, the $C_P(T)$ of q-TIP4P/F water is in semiquantitative agreement with experiments down to $T \approx 240$ K. The maximum in $C_P(T)$, at $P=0.1$ MPa, occurs at 228 K and 216 K in experiments and q-TIP4P/F water, respectively. However, the maxima of $C_P(T)$ in q-TIP4P/F water is much smaller and wider than found in the experiments of Pathak et al.^10^. This is consistent with the estimated location of the LLCP in experiments and in our simulations. The LLCP in real water is estimated to be located at $P_C \approx$ 50–100 MPa and $T_C \approx 220$ K, while in q-TIP4P/F water we find $T_c$ =159 K and $P_c$ = 167 MPa. Accordingly, at $P=0.1$ MPa, experiments are much closer to the corresponding LLCP than in the case of q-TIP4P/F water^6^. Our results for $C_P(T)$ also imply that PIMD and MD simulations of q-TIP4P/F water cannot reproduce the entropy fluctuations observed in real water at low temperatures and pressures.

### Diffusion coefficient

**Figure 6:**
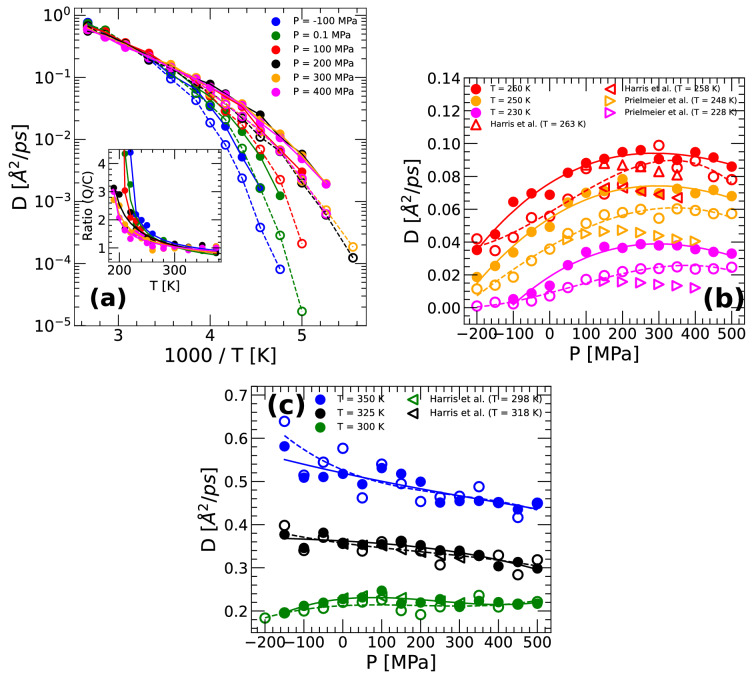
(**a**) Diffusion coefficient of q-TIP4P/F water as function of temperature at selected pressures. Results from RPMD simulations are indicated by solid circles; empty circles are results from classical MD simulations. Inset: ratio of the diffusion coefficients obtained from RPMD and MD simulations shown in the main panel. The inclusion of NQE increases water dynamics upon supercooling. (**b**,**c**) Diffusion coefficients of q-TIP4P/F water as function of pressure obtained from RPMD (solid circles) and classical MD simulations (empty circles); empty triangles correspond to experimental data (left-triangles and right-triangles from Refs.^70,71^, respectively). An anomalous diffusivity maximum exist in q-TIP4P/F water at approximately $T < 300$ K.

We also calculate the self-diffusion coefficient of q-TIP4P/F water *D*(*T*) as function of temperature at selected pressures. To obtain *D*(*T*), we employ the same methodology used in Ref.^46^. Briefly, using the RPMD simulation technique, we first calculate the mean-square displacement (MSD) of the oxygen atoms/ring-polymer centroids. *D*(*T*) is then evaluated from the slope of the MSD at long times, in the so-called diffusive regime. Figure 6a shows the *D*(*T*) of q-TIP4P/F water obtained from RPMD (solid circles) and classical MD (open circles) simulations. At high temperatures, *D*(*T*) is Arrhenius at all pressures studied, i.e.,

5 $$\begin{aligned} D(T) = D_1 exp\left( -\frac{E_A}{k_BT}\right) , \end{aligned}$$

where $D_1$, $E_A$ are constants; $k_B$ is the Boltzmann constant. At low temperatures, the behavior of *D*(*T*) depends on the pressure. Specifically, at high pressures, $P \ge 200$ MPa, in the HDL domain, *D*(*T*) is non-Arrhenius and its behavior is well described by Mode Coupling Theory (MCT), i.e.,

6 $$\begin{aligned} D(T) = D_0 (T - T_{MCT})^{\gamma }, \end{aligned}$$

where $D_0$, $\gamma$, and the MCT temperature $T_{MCT}$ are constants. At low pressures (< 200 MPa), *D*(*T*) also obeys MCT but only down to approximately $T=$ 200–220 K. Upon further cooling, *D*(*T*) seems to exhibit a crossover from non-Arrhenius ($T >$ 200–220 K) to Arrhenius ($T < 200$ K) behavior. An Arrhenius regime at low temperatures can be identified in Fig. 6a for the case $P=-100$ MPa; the cases of $P=100, 0.1$ MPa are less evident due to the limited available data at $T < 200$ K.

The values of *D* from the MD/PIMD simulations are compared with the corresponding experimental values in Fig. 6b,c along different isotherms. At high temperatures, approximately $T>300$ K (Fig. 6c), the values of *D*(*P*) from classical MD and PIMD simulations practically overlap with the available experimental data at all pressures studied. Instead, at $T<300$ K (Fig. 6b), our simulation results predict values of *D*(*P*) that deviate by up to a factor of 4 from the corresponding experimental data, depending on pressure. Interestingly, overall, classical MD simulations are in slightly better agreement with experiments compared to the RPMD simulation results. Not surprising, as shown in the inset of Fig. 6a, the inclusion of NQE increases water diffusivities, particularly upon cooling within the supercooled regime.

One of the main points of Fig. 6b is the presence of an anomalous maximum in the diffusion coefficient of q-TIP4P/F water. Such a *D*-maximum is consistent with experiments and implies that there is a range of temperatures at which *D* increases (anomalously) with increasing *P*.

### Phase diagram

**Figure 7:**
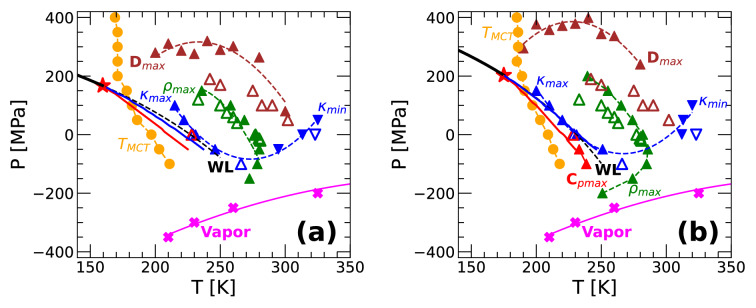
Phase diagram of q-TIP4P/F H$_2$O obtained from (**a**) PIMD/RPMD and (**b**) classical MD simulations. The black solid and dashed lines are, respectively, the LLPT and Widom lines obtained from the TSEOS; the filled red star is the LLCP from the TSEOS. The red and blue solid lines are the $C_P$-maxima and $\kappa _T$-maxima line from the TSEOS. The magenta crosses represent the vaporization line where the liquid spontaneously cavitates during the computer simulations. The green up-triangles are the density maxima. Blue up and down triangles represent, respectively, the maxima and minima in the isothermal compressibilities; red triangles indicate the maxima in $C_P$. Brown up-triangles are the maxima in the diffusion coefficient; orange circles represent the MCT temperature (see Eq. 5). Experimental data are shown by empty triangles^5,19,71,78–80^; filled symbols are results from MD/PIMD simulations. The estimated location of the LLCP based on experiments is ($T_c = 220$ K, $P_c =$ 50–100 MPa)^12^.

Figure 7a,b show, respectively, the phase diagram of q-TIP4P/F water obtained from PIMD and classical MD simulations. These phase diagrams include the LLCP, coexistence line, $C_P$-maxima, $\kappa _T$-maxima, and Widom line calculated from the TSEOS. Also included are the lines of $\rho$-maxima, *D*-maxima, $C_P$-maxima, $\kappa _T$-maxima, and $\kappa _T$-minima obtained from our MD/PIMD simulations. We also show the maxima/minima of these properties reported in experiments, where available. The liquid-vapor boundary lines shown in Fig. 7a,b correspond to the conditions at which spontaneous cavitation occurs during our computer simulation.

**Figure 8:**
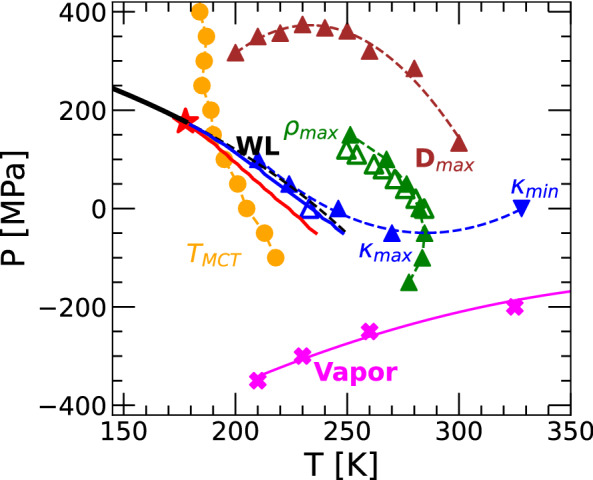
Same as Fig. 7 for the case of D$_2$O. Results are from PIMD/RPMD simulations using the q-TIP4P/F water model. The empty green-up triangles are experimental maxima densities from Ref.^81^. The empty blue-up triangle is the isothermal compressibility maxima from Ref.^5^. The estimated location of the LLCP based on experiments is ($T_c = 230$ K, $P_c = 50$ MPa)^50^.

The phase diagrams of q-TIP4P/F water resulting from the classical MD and PIMD simulations are qualitatively similar. As found previously in ST2^42^ and TIP4P/2005 water^15^, the $C_P$ and $\kappa _T$-maxima lines obtained from the TSEOS originate at the LLCP and deviate from each other at higher temperatures. The $\kappa _T$-maxima line (blue up-triangles) connects smoothly with the $\kappa _T$-minima line (blue down-triangles). In addition, as shown in Ref.^35^, the point in Figs. 7 and 8 where the $\rho$-maxima line has infinite slope is located on the $\kappa _T$-extrema line, at which $(\partial \kappa _T/ \partial T)_P = 0$. The $\rho$-maxima line has a nose shape. In particular, our simulations suggest that, at low pressures, the $\rho$-maxima line is re-entrant and deviates from the liquid-vapor boundary line [see, in particular, Fig. 7b]. This implies that the ’re-entrant spinodal’ hypothesis scenario proposed to explain water anomalous behavior is not supported by MD/PIMD simulations of q-TIP4P/F water. Hence, our results are consistent with Refs.^7,72^, where computer simulations performed using the ST2 and TIP4P water model found that the spinodal line is also not re-entrant. We note that Fig. 7a,b also include available experimental data. When compared with experiments, both classical MD and PIMD reproduce correctly the location of the $\kappa _T$-maxima and minima lines; the PIMD simulation results reproduce slightly better the location of the $\rho$-maxima line.

Regarding the *D*-maxima line, both MD and RPMD simulations overestimate the corresponding pressures relative to the experiments, with the RPMD simulations performing slightly better. We also include in Fig. 7a,b the MCT temperatures extracted from Fig. 6a. The experimental MCT temperature at $P=0.1$ MPa is $T_{MCT}=221$ K^73^ and hence, this temperature is underestimated in both MD and RPMD simulations.

Overall, the results from classical MD simulations in Fig. 7b are consistent with the TSEOS. Accordingly, one may conclude that the reported location of the LLCP based on the TSEOS is robust in the case of classical MD simulations. For example, the $\kappa _T$-maxima from MD simulations and from the TSEOS (blue triangles and blue solid lines) overlap; similarly, the corresponding $C_P$-maxima lines (red triangles and red solid lines) also overlap. In addition, we find that the $T_{MCT}(P)$ line shows a sudden change in slope at the intersection with the Widom line. This is consistent with the view that the Widom line separates the LDL-like liquid at low pressures from the HDL-like liquid at high pressures. The sharp crossover in $T_{MCT}(P)$ is reminiscent of the glass transition temperature of water as a function of pressure which shows a sudden change as the system evolves from LDL (low pressure) to HDL (high pressure)^31,74,75^.

In the case of PIMD simulations (Fig. 7a), the $\kappa _T$-maxima line obtained from the TSEOS (blue solid line) is located at slightly lower pressure relative to the PIMD simulation results (blue up-triangles). Similarly, the Widom line predicted by the TSEOS is located at a pressure slightly lower than the pressure at which the slope of $T_{MCT}(P)$ suddenly changes. Hence, in the case of PIMD simulations for water, the reported location of the LLCP may shift slightly if additional data points at $T < 180$ K are considered in the TSEOS calculation. Additional PIMD simulations are also needed to improve the determination of the $\kappa _T$-maxima line at low temperatures.

The similarities in the phase diagrams of Fig. 7a,b imply that, at least for the q-TIP4P/water model, the LLPT hypothesis scenario remains a solid, viable explanation of water anomalous behavior even if NQE (i.e., atoms delocalization) are included. This is important since (i) the LLCP scenario has been tested only in classical MD simulations and, mostly, rigid water models, and (ii) the location of the LLCP is extremely sensitive to small variations in the water model considered (e.g., small changes in the partial charges of the H and O atoms can easily shift the location of the LLCP to (*P*, *T*) conditions that are physically inaccessible to the liquid state; see, e.g., Refs.^76,77^). Overall, including NQE shifts the location of the LLCP, LLPT line, and maxima/minima lines towards lower temperatures (see also Ref.^47,48^).

So far, our discussion has been centered on H$_2$O. The same analysis presented here for H$_2$O was done for the case of D$_2$O by performing PIMD simulations using the q-TIP4P/F model. The analogous to Figs. 1, 2, 3, 4, 5 and 6 are included in Figs. S4–S8 of the SI. Here, we only discuss the phase diagram of q-TIP4P/F D$_2$O; see Fig. 8. The phase diagram of D$_2$O is qualitatively similar to the phase diagram of H$_2$O. The LLCP in q-TIP4P/F D$_2$O is located at ($\rho _c=1.13$ g/cm$^3$, $P_c=176$ MPa, $T_c=177$ K). Relative to q-TIP4P/F H$_2$O, the LLCP in D$_2$O is shifted by ($\Delta \rho _c \approx 0.11$ g/cm$^3$, $\Delta P_c \approx 9$ MPa, $\Delta T_c \approx 18$ K). Accordingly, our results indicate that isotope substitution in water can play an important role in the phase behavior of low-temperature and supercooled water. These values of $\Delta T_c$ and $\Delta P_c$ are consistent with the locations of the LLCP in H$_2$O and D$_2$O *estimated* by Mishima and Stanley from decompression-induced experiments of ice IV^49,50^ where ($\Delta P_c\approx 50$ MPa, $\Delta T_c\approx 10$ K).

## Summary and discussion

We performed classical MD, PIMD, and RPMD simulations of H$_2$O using the q-TIP4P/F model over a wide range of temperatures and pressures. At supercritical temperatures, most properties studied are practically insensitive to whether one employs classical MD and PIMD simulations ($-100 \le P \le 500$ MPa). Specifically, the $\rho (T)$ and $\kappa _T(T)$ obtained from classical MD or PIMD simulations overlap (within error bars) down to $T \approx 225$ K and $T \approx 200$ K, respectively (Fig. 1 and Fig. S2 of the SI). In the case of $C_P(T)$, the quantitative values vary for classical MD and PIMD results, but this is expected to occur due to NQE^68^. Nonetheless, the qualitative behavior of $C_P(T)$ is independent on whether NQE are included or excluded. Similarly, the behavior of *D*(*T*) is not affected whether one employs classical MD or RPMD simulations down to $T \approx 260$ K (Fig. 6a). Relative to the classical MD simulations, including NQE (RPMD simulations) increases the values of *D*(*T*) at $T<260$ K (inset of Fig. 6a). In both cases, the *D*(*T*) from computer simulations are in good agreement with experiments where data is available (Fig. 6b,c).

Deviations between MD and PIMD simulations become noticeable at approximately $P=$ 100–200 MPa and $T<225$ K. Our results strongly indicate that at these thermodynamic conditions, q-TIP4P/F water exhibits a LLCP. Using a two-state equation of state, we estimate that the LLCP is located at $(\rho _c=1.03$ g/cm$^3$, $P_c=203$ MPa, $T_c=175$ K) when NQE are not included (classical MD); including NQE (PIMD simulations) shifts the location of the LLCP to $(\rho _c=1.02$ g/cm$^3$, $P_c=167$ MPa, $T_c=159$ K); see Fig. 1c,d. Consistent with the existence of a LLCP, our study shows the presence of loci of maxima in $C_P$ and $\kappa _T$ in the P–T phase diagram of q-TIP4P/F water. These anomalous maxima lines, together with the loci of maxima in *D* and $\rho$ are included in Fig. 7. We stress that the location of the LLCPs reported in this work are estimations provided by the TSEOS. Our estimation of the LLCP for H$_2$O is based on PIMD simulations using $n_b = 32$ beads per ring-polymer. While PIMD simulations with $n_b > 32$ are computationally expensive, additional PIMD simulations employing $n_b > 32$ beads per ring-polymer are desirable at low temperatures in order to obtain a more precise estimation of the LLCP location in H$_2$O (q-TIP4P/F water). While our results conclusively show that the LLCP in H$_2$O shifts to lower T when NQE are included, obtaining the exact values of ($\rho _c,~T_c,~P_c$) may require additional data at $T < 180$ K (particularly for the case of PIMD simulations of H$_2$O where $T_c$ is low).

Overall, our results for H$_2$O (e.g., Fig. 7) are consistent with previous classical computer simulations of water using the (rigid) ST2^42^ and TIP4P/2005^15^ water models. It follows that the present study validates the LLPT hypothesis for water to the case where NQE are included. We note, however, that the LLCP in q-TIP4P/F water, as well as in ST2, TIP4P/2005, and TIP4P/Ice water, is located at pressures and temperatures that are off compared to the experimental predictions^6,46^. This provides a *thermodynamic* explanation of why these water models are unable to reproduce the sharp increase in $C_P(T)$ and $\kappa _T(T)$ observed in experiments at $P=0.1$ MPa^5,10^. Specifically, these water models predict that $P_c > 150$ MPa, while $P_c \approx 50 - 100$ MPa estimated from experiments^1,50^. Accordingly, computer simulations show a mild increase in $\kappa _T$ and $C_P$, relative to experiments, upon isobaric cooling at P = 0.1 MPa. We note that the $C_P(T)$ and $\kappa _T(T)$ quantify the fluctuations in entropy and volume, respectively. Hence, from a *microscopic* point of view, the weak increase of $\kappa _T$ and $C_P$ upon isobaric cooling at *P* = 0.1 MPa is due to the inability of current water models (ST2, TIP4P/2005, TIP4P/Ice, etc) to reproduce the anomalously large fluctuations (in entropy and volume) of real water in the supercooled regime. At least for the q-TIP4P/F model studied, the inclusion of NQE (quantum fluctuations due to atoms delocalization) is not sufficient to reproduce the anomalously large fluctuations (in entropy and volume) of real water at low temperatures. Accordingly, additional sources of fluctuations may be missed in rigid (e.g., TIP4P/2005, ST2) as well as flexible water models, such as q-TIP4P/F model.

We also performed extensive PIMD simulations of heavy water using the q-TIP4P/F model. The results are summarized in the phase diagram of Fig. 8 (see also the SI). The PIMD simulations confirm that isotope substitution has minor effects on the properties of water. While the phase diagram of D$_2$O is qualitatively identical to the phase diagram of H$_2$O, the location of the corresponding LLCP differ. Specifically, calculations based on the TSEOS, applied to PIMD data at $T \ge 190$ K, predict that in the case of D$_2$O, ($\rho _c=1.13$ g/cm$^3$, $P_c=176$ MPa, $T_c=177$ K) which represents a non-negligible shift relative to H$_2$O ($\Delta \rho _c=0.11$ g/cm$^3$, $\Delta P_c \approx 9$ MPa, $\Delta T_c \approx 18$ K). This is important since computer simulations of water-like models show that the introduction of NQE can indeed shift considerably the location of the LLCP^47,48^. In particular, the differences in the relative values of ($\rho _c$, $P_c$, $T_c$) between q-TIP4P/F H$_2$O and D$_2$O are somewhat consistent with the predictions from experiments in glassy water ($\Delta P_c=50$ MPa, $\Delta T_c=10$ K)^49,50^ and with the relative location of the $\kappa _T$-maximum at 1 bar^10^. The present study shows that many questions previously addressed in computational studies of supercooled water at low temperatures, using classical water models, are accessible via PIMD simulations. For example, it would be interesting to explore the relationship between dynamics and structure of water at low temperatures and how the Stokes–Einstein and Stokes–Einstein–Debye relationships are affected by isotope substitution^82–85^.

## Supplementary Information

Supplementary Information.

## Data Availability

All study data are included in the article and/or SI.
