# Supplementary material for: Evidence of a liquid–liquid phase transition in H2O and D2O from path-integral molecular dynamics simulations
Source: Sci Rep. 2022 Apr 9;12:6004. doi: 10.1038/s41598-022-09525-x (PMC8994788; doi:10.1038/s41598-022-09525-x)
Supplement: Supplementary file 1 — Supplementary Information. [file 41598_2022_9525_MOESM1_ESM.pdf]

Supplementary Information for 'Evidence of a Liquid-liquid Phase Transition in H_2O and D_2O from Path-Integral Molecular Dynamics Simulations'

Ali Eltareb[1,3], Gustavo E. Lopez[2,4], and Nicolas Giovambattista[1,3,4]

[1]*Department of Physics, Brooklyn College of the City University of New York, Brooklyn, New York 11210, United States*

[2]*Department of Chemistry, Lehman College of the City University of New York, Bronx, New York 10468, United States*

[3]*Ph.D. Program in Physics, The Graduate Center of the City University of New York, New York, NY 10016*

[4]*Ph.D. Program in Chemistry, The Graduate Center of the City University of New York, New York, NY 10016*

I. COMPUTER SIMULATIONS

We perform classical molecular dynamics (MD) and path-integral MD computer simulations (PIMD) of H_2O at the (T, P) indicated in Fig. S1. The state points studied using PIMD simulations of D_2O are also included. In all cases, the q-TIP4P/F water model [1] is employed.

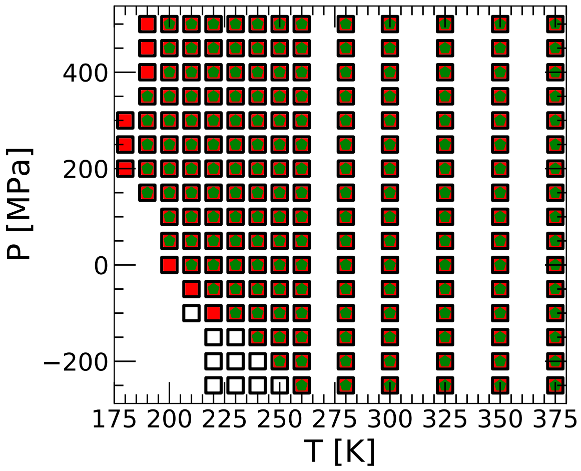

FIG. S1. State points studied in this work via classical MD and PIMD simulations of H_2O and D_2O using the q-TIP4P/F model. Black and red squares indicate, respectively, the state points studied using classical MD and PIMD simulations of H_2O. Green pentagons represent state points studied using PIMD simulations of D_2O.

II. ISOTHERMAL COMPRESSIBILITY AND ISOBARIC HEAT CAPACITY OF H_2O

Here, we address two points made in the main manuscript regarding H_2O. Specifically, (i) the isothermal compressibility of H_2O obtained from classical MD and PIMD simulations are identical, within error bars at most conditions studied; see Fig. S2. Again, this implies that nuclear quantum effects (NQE) (i.e., the delocalization of water atoms) play a minor role at approximately $T \geq 200$ K ($-100 \leq P \leq 400$ MPa), at least, in q-TIP4P/F water.

(ii) The isobaric heat capacity $C_P(T)$ is sensitive to the fitting procedure employed. In a

previous work [2], we calculate $C_P(T)$ by using the expression,

$$C_P(T) = \left(\frac{\partial H}{\partial T}\right)_{N,P} \tag{1}$$

where $H(T)$ was obtained by fitting the values for $H(T)$ obtained directly from the computer simulations using a fourth-order polynomial. As pointed out in the main manuscript, this procedure leads to a $C_P(T)$ that does not exhibit a maximum at low temperatures. As shown in Fig. S3(a), a fourth-order polynomial fits very well the values of $H(T)$ of H_2O obtained directly from our PIMD simulations. While the solid lines in Fig. S3(a) (fourth-order polynomial) and Fig. 4(a) of the main manuscript (TSEOS) both fit remarkably well the values of $H(T)$ from the PIMD simulations, the corresponding values of $C_P(T)$ obtained from Eq. 1 are qualitatively different. The C_P-maxima in Fig. 4(a) of the main manuscript are absent in the $C_P(T)$ of Fig. S3(b). Although $C_P(T)$ increases upon cooling at low pressures (Fig. S3(b)), as found in experiments, the rate at which $C_P(T)$ increases in our PIMD simulations is severely underestimated. At high pressures, experiments show that $C_P(T)$ decreases upon cooling, while our PIMD simulations show that $C_P(T)$ increases as the temperature decreases.

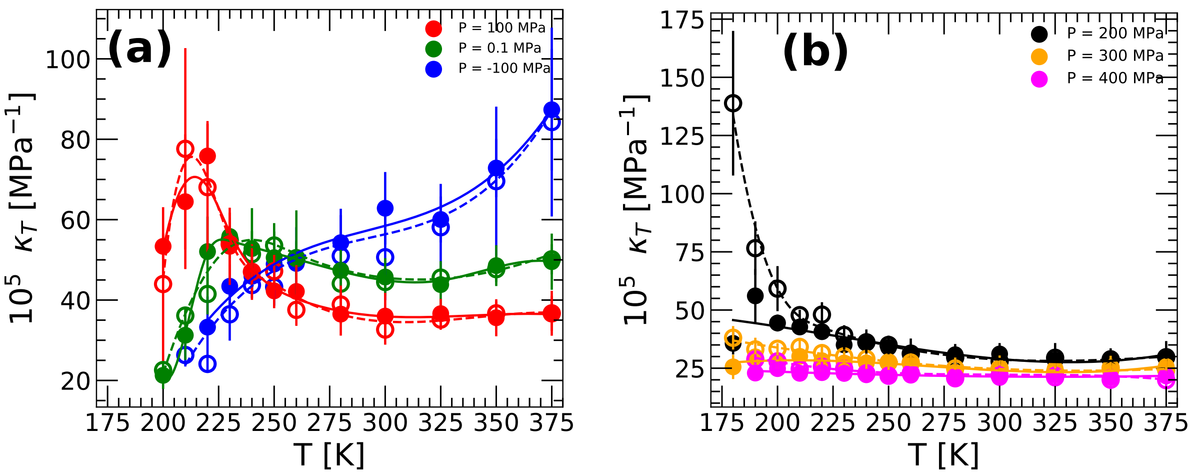

FIG. S2. Isothermal compressibility of H_2O from classical MD (empty circles) and PIMD simulations (solid circles; from Figs. 3(a) and 3(b) of the main manuscript) at selected pressures, above and below the LLCP pressure. The values of $\kappa_T(T)$ obtained from MD and PIMD simulations overlap within error bars. Lines are guides to the eye.

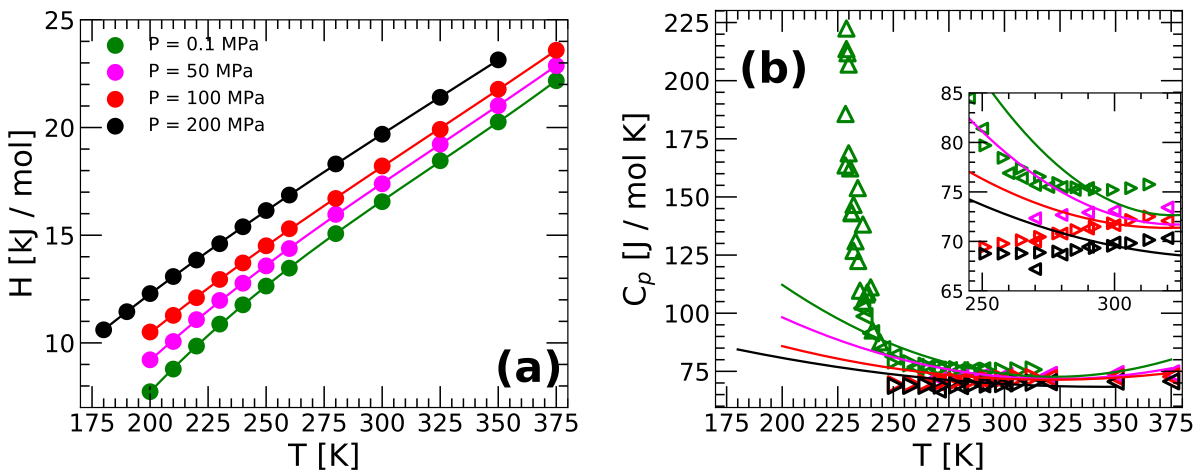

FIG. S3. (a) Enthalpy $H(T)$ of H_2O obtained from PIMD simulations at selected pressures (solid circles; from Fig. 4(a)). Lines are fits using a fourth-order polynomials. (b) Heat capacity $C_P(T)$ of H_2O obtained by differentiation of the fourth-order polynomials shown in (a). Results from PIMD simulations are indicated by solid lines; symbols are the corresponding experimental data (up-triangles are from Refs. [3]; left-triangles are from Refs. [4, 5]; right-triangles are from Refs. [6]). At low pressures, the fitting procedure employed here to calculate $H(T)$ leads to an increasing $C_P(T)$ upon cooling, in qualitative agreement with experiments, but there is no C_P-maximum at low temperatures, in disagreement with experiments. At high pressures, the behavior of $C_P(T)$ found in experiments and simulations is qualitatively different.

III. RESULTS FOR D_2O FROM PIMD SIMULATIONS

Next, we include results obtained from PIMD simulations of D_2O. Specifically, Fig. S4 shows the density of D_2O as function of temperature together with the corresponding TSEOS. The pressure and potential energy of the system along isotherms are included in Fig. S5. The $\kappa_T(T)$, $H(T)$, and $C_P(T)$ are shown, respectively, in Figs. S6, S7a, and S7b. The diffusion coefficient of D_2O is included in Fig. S8.

Overall, the results presented in Figs. S4-S8 for D_2O are qualitatively identical to those shown in Figs. 1-6 of the main manuscript for the case of H_2O. The main effect of isotope substitution H→D in water is mostly to shift the phase diagram of liquid water to higher temperatures by > 10 K; see Fig. S9.

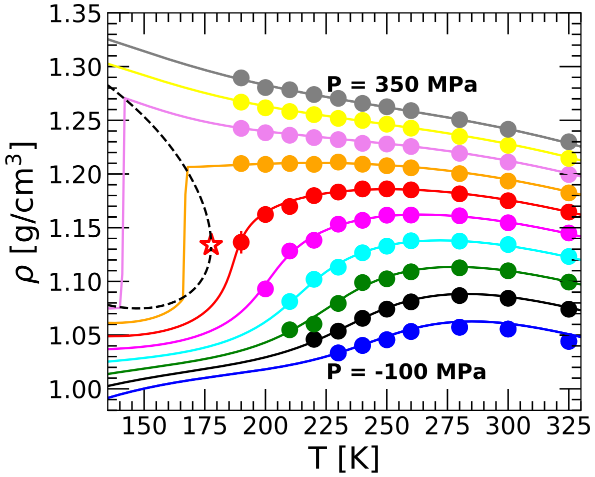

FIG. S4. Density of D_2O obtained from PIMD simulations using the q-TIP4P/F water model. Same as Fig. 1(d) of the main manuscript for the case of D_2O.

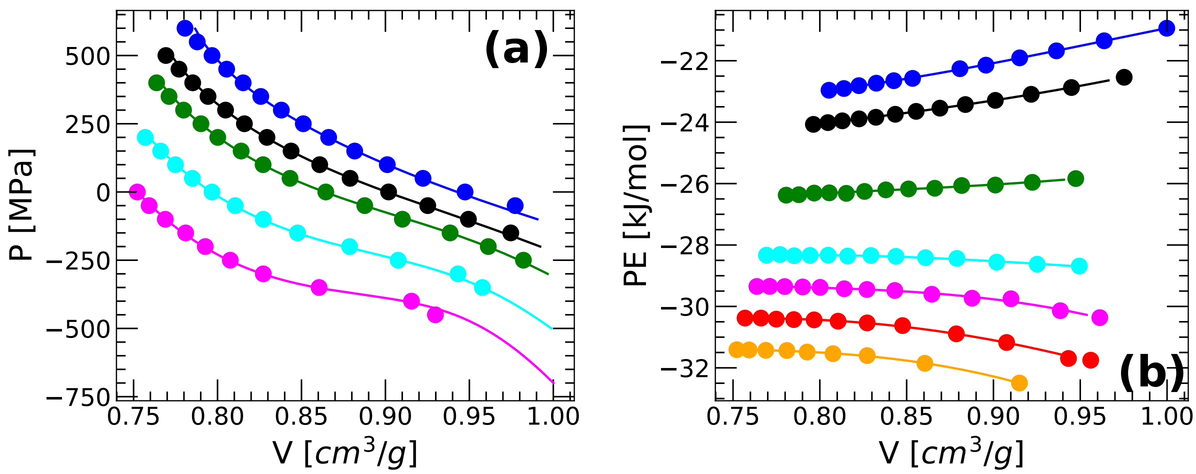

FIG. S5. (a) Pressure and (b) potential energy of D_2O as function of volume obtained from PIMD simulations of q-TIP4P/F water. Same as Figs. 2(b) and 2(c) of the main manuscript for the case of D_2O. In (a) isotherms correspond to (top to bottom) $T = 300,\ 260,\ 240,\ 220,\ 200$ K and are shifted by $\delta P = 100,\ 0,\ -100,\ -300,\ -500$ MPa, respectively. Temperatures in (b) are (top to bottom) $T = 375,\ 350,\ 300,\ 260,\ 240,\ 220,\ 200$ K.

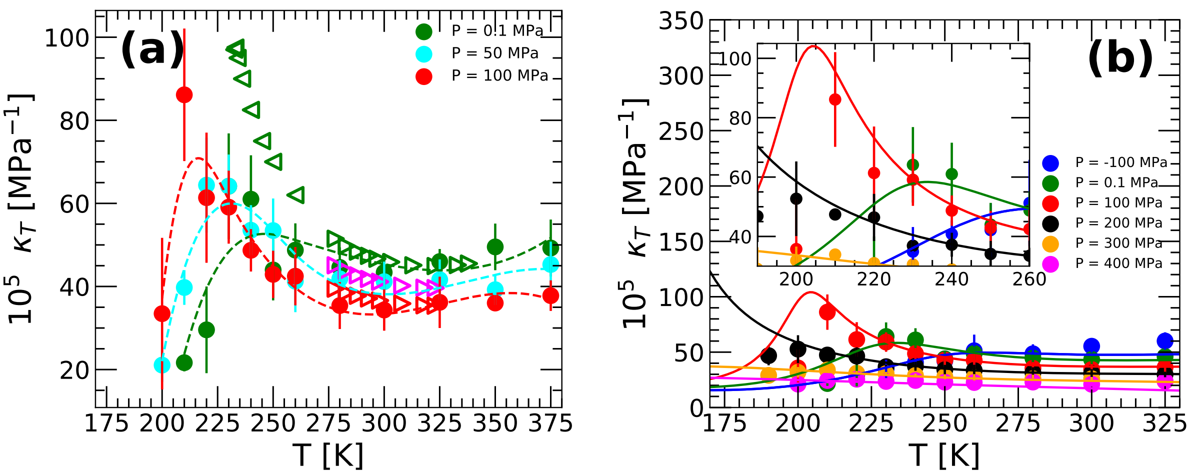

FIG. S6. Isothermal compressibility of D_2O from PIMD simulations using the q-TIP4P/F water model (compare with Fig. 3 of the main manuscript for H_2O). (a) Comparison between PIMD simulations (solid symbols) and experiments (empty symbols) [7–9]; dashed-lines are guide to the eye. (b) Comparison between PIMD simulations (solid symbols) and the corresponding predictions from the TSEOS (solid lines).

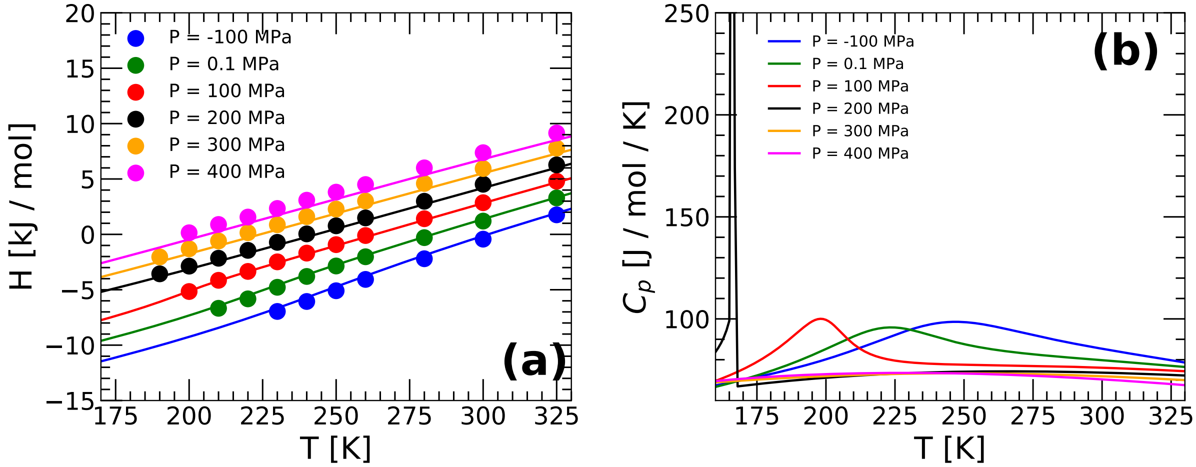

FIG. S7. (a) Enthalpy of D_2O from PIMD simulations using the q-TIP4P/F model (solid symbols). Lines are the predictions from the TSEOS. Same as in Fig. 4(a) of the main manuscript for the case of D_2O. (b) Isobaric heat capacity obtained from the TSEOS enthalpies shown in (a) by differentiation with respect to T. Same as Fig. 5(a) of the main manuscript for the case of D_2O.

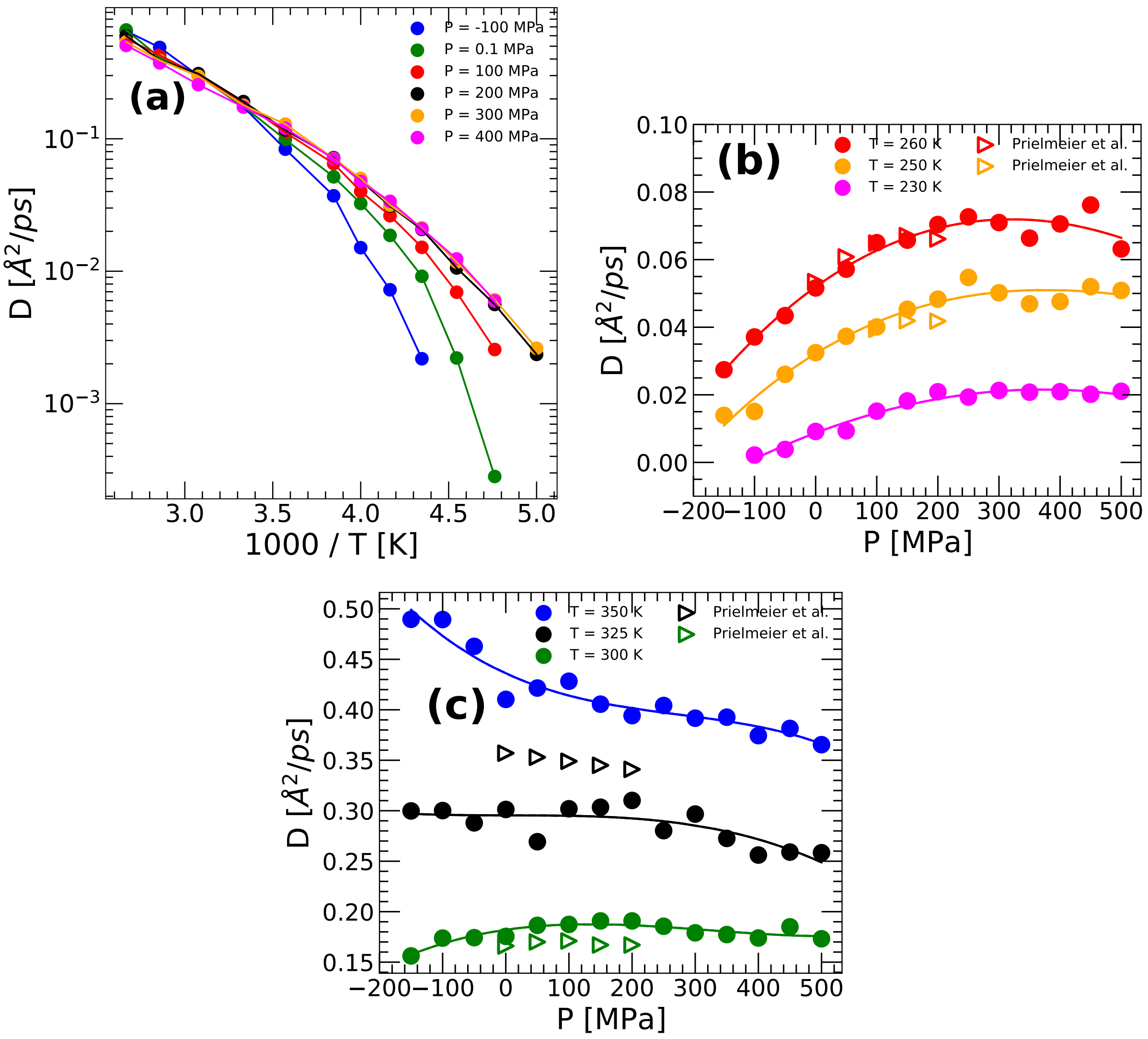

FIG. S8. (a) Diffusion coefficient of D_2O as a function of temperature from PIMD simulations using the q-TIP4P/F model. Same as Fig. 6(a) from the main manuscript for the case of D_2O. (b)(c) Diffusion coefficient of D_2O as a function of pressure; solid and empty symbols are results from PIMD simulations and experiments [10], respectively. Same as Figs. 6(b)-(c) from the main manuscript for the case of D_2O.

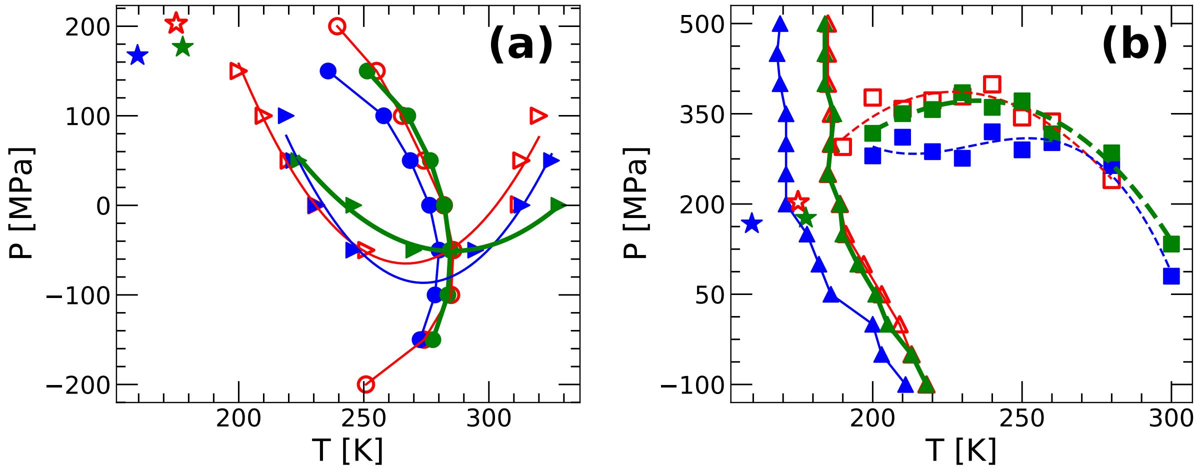

FIG. S9. NQE and isotope substitution effects on the phase diagram of q-TIP4P/F water. (a) Loci of ρ-maxima (circles), C_P-maxima (left triangles), and κ_T-maxima and minima (right-triangle); the stars represent the LLCP. Results for H_2O obtained from classical MD and PIMD simulations are shown with empty red and solid blue symbols, respectively; results for D_2O are shown by solid green symbols. (b) Locus of D-maxima (squares) and Mode Coupling temperature, $T_{MCT}(P)$ (up-triangles). NQE due to atoms delocalization tend to shift the phase diagram of water to lower T (red and blue symbols/lines). Similar effects follow due to isotope substitution ($D \rightarrow H$) (green and blue symbols/lines).

IV. RESULTS FOR H_2O FROM PIMD SIMULATIONS USING n_b = 32, 72

In Ref. [2], we performed PIMD simulations using n_b = 32, 72, and 128 beads per ring-polymer at P = 0.1 MPa and over a wide range of temperatures. It was shown that the thermodynamic properties of H_2O and D_2O (density, isothermal compressibility, dielectric constant) and their dynamical properties (diffusion coefficient) to be sufficiently converged with $n_b \geq 32$ beads per ring-polymer. Small differences in the enthalpy and heat capacity remain. In order to ensure that our PIMD results in the manuscript are converged and do not depend on n_b, we performed PIMD simulations using $n_b = 72$ for the isobars $P = 250$ MPa and $P = 500$ MPa. The density $\rho(T)$, isothermal compressibility $\kappa_T(T)$, enthalpy $H(T)$, and diffusion coefficient $D(T)$ are shown in Fig. S10 for $n_b = 32, 72$. The thermodynamic and dynamical properties at these pressures also converged with $n_b = 32$; differences between the cases $n_b = 32$ and $n_b = 72$ were negligible, consistent with our previous conclusions [2]. These results support that the $\rho - T$ phase diagrams reported for H_2O and D_2O are robust and do not depend on n_b. Again, as discussed in Ref. [2], small differences in $H(T)$ are observable at low T.

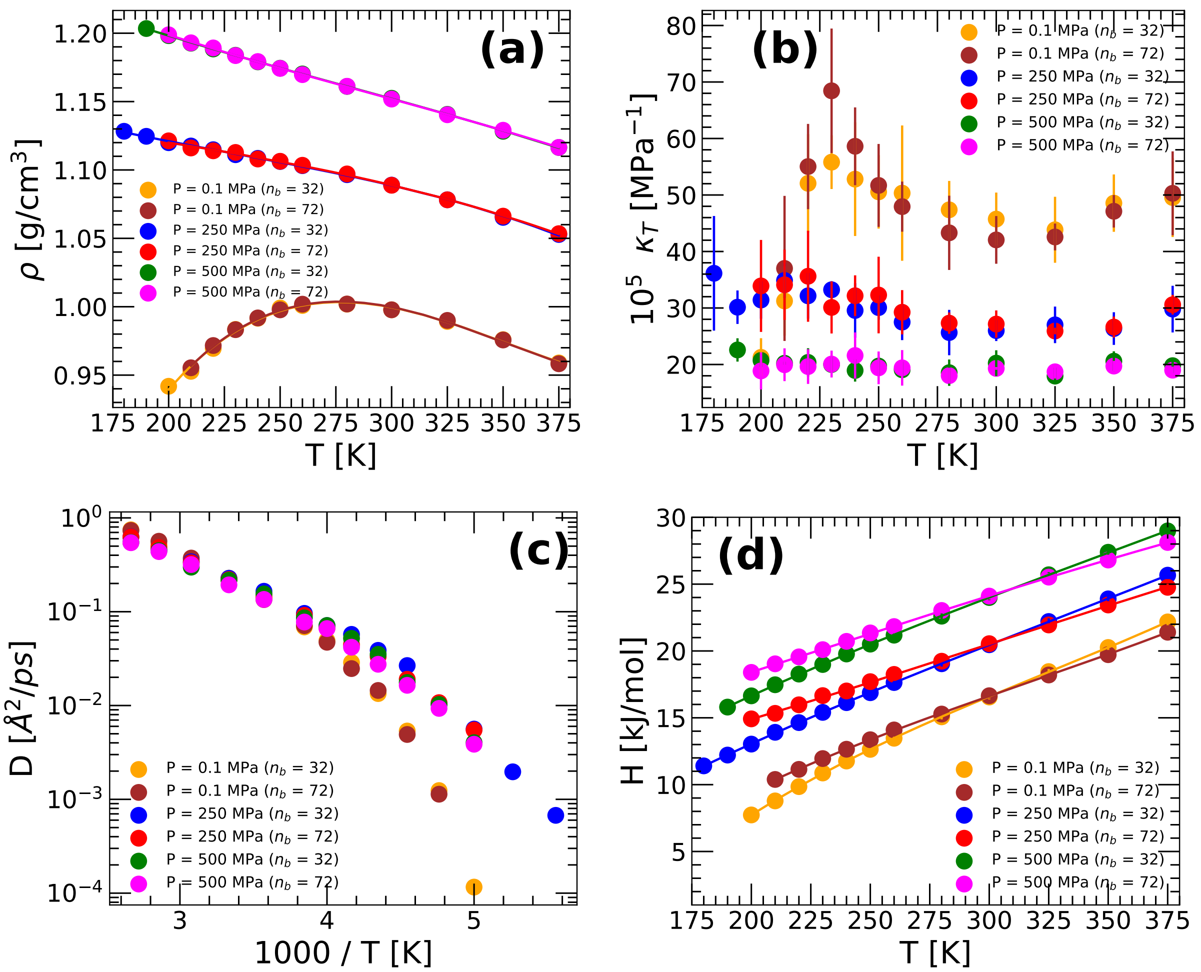

FIG. S10. (a) Density, (b) isothermal compressbility, (c) diffusion coefficient, and (d) enthalpy of H_2O as a function of temperature from PIMD simulations using the q-TIP4P/F model and for $n_b = 32,\ 72$. For $P = 0.1$ MPa, results for $n_b = 72$ are from Ref. [2].

V. TWO-STATE EQUATION OF STATE

The TSEOS provides an expression for the Gibbs free energy of the system, $G(N, P, T)$, in terms of 20 fitting parameters; details can be found in Refs. [11–13]. In this work, we follow the same procedure of Gartner *et al.* [11] paper. The software provided in Ref. [11] to obtain the TSEOS was used here with minor modifications to manipulate our data. The 20 parameters that fit our MD and PIMD simulation data are given in Tables S1-S3. Using these parameters, we calculate the $G(N, P, T)$ and hence, all thermodynamic properties, predicted by the TSEOS.

The pressures used to obtain the TSEOS are $-50 \leq P \leq 350$ MPa, which expand below and above the LLCP pressure. The temperatures used to obtain the TSEOS reported in the manuscript are $180 \leq T \leq 325$ K for H_2O and $190 \leq T \leq 325$ K for D_2O. We also considered different temperature intervals (i) $T \leq 300$ K and (ii) $T \leq 350$ K in order to check the robustness of our estimation for the LLCP. The LLCP obtained using the different temperature intervals overlap within error bars with our original estimation of the LLCP reported in the manuscript (for the case of $T \leq 325$ K). The fitting parameters for the TSEOS based on the MD and PIMD simulation data are given in Table S1-S3.

To estimate the errors in (T_c, P_c, ρ_c), we used 50 different set of starting parameters and calculate the corresponding (T_c, P_c, ρ_c). Standard deviations in (T_c, P_c, ρ_c) are included in Tables S1-S3. Briefly, we find that P_c can vary by about $10 - 25$ MPa, T_c varies by about $3 - 10$ K, and ρ_c can vary by about 0.01 g/cm^3.

	$180 \leq T \leq 300$ K	$180 \leq T \leq 325$ K	$180 \leq T \leq 350$ K
ρ_c	1.03 (0.01)	1.03 (0.01)	1.03 (0.01)
P_c	203 (4)	203 (4)	203 (6)
T_c	175 (1)	175 (2)	175 (2)
λ	1.511	1.470	1.515
a	0.403	0.417	0.404
b	-0.266	-0.299	-0.266
w_0	0.212	0.206	0.218
c_{00}	2.082	2.086	2.089
c_{01}	-0.009	-0.008	-0.007
c_{02}	0.114	0.112	0.107
c_{11}	-6.75	-7.328	-7.363
c_{20}	-0.034	-0.051	-0.048
c_{12}	0.168	0.174	0.191
c_{21}	1.291	2.485	2.635
c_{30}	-0.078	-0.031	-0.026
c_{22}	-0.041	-0.036	-0.057
c_{31}	0.331	-0.449	-0.555
c_{40}	0.019	0.037	0.025
c_{23}	0.066	0.024	0.014
c_{32}	0.001	-0.026	-0.011
c_{33}	-28.826	-29.0	-28.966

TABLE S1: LLCP coordinates and fitting parameters of the TSEOS for the case of H_2O from classical MD simulations considering different temperature intervals. Numbers in parenthesis are the standard deviations. T_c is in Kelvin, P_c is in MPa, and ρ_c is in g/cm^3.

	$180 \leq T \leq 300$ K	$180 \leq T \leq 325$ K	$180 \leq T \leq 350$ K
ρ_c	1.02 (0.01)	1.02 (0.01)	1.02 (0.01)
P_c	165 (11)	167 (9)	163 (8)
T_c	159 (7)	159 (6)	164 (4)
λ	1.128	1.058	1.174
a	0.502	0.542	0.446
b	-0.175	-0.264	-0.224
w_0	0.108	0.074	0.113
c_{00}	1.907	1.930	1.821
c_{01}	-0.021	-0.023	-0.018
c_{02}	-0.028	-0.027	0.009
c_{11}	-4.99	-4.922	-5.305
c_{20}	0.04	0.042	0.02
c_{12}	0.176	0.214	0.179
c_{21}	1.367	1.387	1.783
c_{30}	-0.049	-0.095	-0.068
c_{22}	-0.028	-0.052	-0.040
c_{31}	-0.186	-0.236	-0.364
c_{40}	-0.018	0.001	0.005
c_{23}	-0.006	0.034	0.024
c_{32}	0.024	0.005	0.001
c_{33}	6.315	6.397	6.306

TABLE S2: Same as Table S1 for H_2O from PIMD simulations.

	$190 \leq T \leq 300$ K	$190 \leq T \leq 325$ K	$190 \leq T \leq 350$ K
ρ_c	1.13 (0.01)	1.13 (0.01)	1.13 (0.01)
P_c	175 (2)	176 (2)	176 (4)
T_c	179 (1)	177 (3)	178 (2)
λ	1.111	1.084	1.084
a	0.403	0.430	0.418
b	-0.242	-0.277	-0.253
w_0	0.111	0.103	0.101
c_{00}	1.615	1.644	1.631
c_{01}	-0.021	-0.020	-0.021
c_{02}	-0.01	-0.015	-0.027
c_{11}	-4.254	-4.416	-4.739
c_{20}	0.041	0.031	0.031
c_{12}	0.179	0.219	0.234
c_{21}	0.595	0.947	1.398
c_{30}	-0.14	-0.118	-0.106
c_{22}	-0.006	-0.044	-0.056
c_{31}	0.135	-0.107	-0.313
c_{40}	0.014	0.017	0.013
c_{23}	0.081	0.062	0.050
c_{32}	-0.006	-0.009	-0.006
c_{33}	-3.477	-3.576	-3.625

TABLE S3: Same as Table S1 for D_2O from PIMD simulations.

[1] S. Habershon, T. E. Markland, and D. E. Manolopoulos, J. Chem. Phys. **131**, 024501 (2009).

[2] A. Eltareb, G. E. Lopez, and N. Giovambattista, Phys. Chem. Chem. Phys. **23**, 6914 (2021).

[3] H. Pathak, A. Späh, K. Amann-Winkel, F. Perakis, K. K. H. Kim, and A. Nilsson, Mol. Phys. **117**, 3232 (2019).

[4] C. Angell, W. Sichina, and M. Oguni, J. Phys. Chem. **86**, 998 (1982).

[5] W. Wagner and A. Pruß, J. Phys. Chem. Ref. Data **31**, 387 (2002).

[6] J. Troncoso, J. Chem. Phys. **147**, 084501 (2017).

[7] K. H. Kim, A. Späh, H. Pathak, F. Perakis, D. Mariedahl, K. Amann-Winkel, J. A. Sellberg, J. H. Lee, S. Kim, J. Park, *et al.*, Science **358**, 1589 (2017).

[8] F. J. Millero and F. K. Lepple, J. Chem. Phys. **54**, 946 (1971).

[9] C.-T. A. Chen and F. J. Millero, J. Chem. Phys. **75**, 3553 (1981).

[10] F. Prielmeier, E. Lang, R. Speedy, and H.-D. Lüdemann, Berichte der Bunsengesellschaft für physikalische Chemie **92**, 1111 (1988).

[11] T. E. Gartner, L. Zhang, P. M. Piaggi, R. Car, A. Z. Panagiotopoulos, and P. G. Debenedetti, Proc. Natl. Acad. Sci. **117**, 26040 (2020).

[12] R. S. Singh, J. W. Biddle, P. G. Debenedetti, and M. A. Anisimov, J. Chem. Phys. **144**, 144504 (2016).

[13] J. W. Biddle, R. S. Singh, E. M. Sparano, F. Ricci, M. A. González, C. Valeriani, J. L. Abascal, P. G. Debenedetti, M. A. Anisimov, and F. Caupin, J. Chem. Phys **146**, 034502 (2017).
